# Glioma Grading via Analysis of Digital Pathology Images Using Machine Learning

**DOI:** 10.3390/cancers12030578

**Published:** 2020-03-02

**Authors:** Saima Rathore, Tamim Niazi, Muhammad Aksam Iftikhar, Ahmad Chaddad

**Affiliations:** 1 Center for Biomedical Image Computing and Analytics, University of Pennsylvania, Philadelphia, PA 19104, USA; 2 Department of Radiology, Perelman School of Medicine, University of Pennsylvania, Philadelphia, PA 19104, USA; 3 Lady Davis Institute for Medical Research, McGill University, Montreal, QC H3S 1Y9, Canada; tamim.niazi@mcgill.ca (T.N.); ahmad.chaddad@mail.mcgill.ca (A.C.); 4 Department of Computer Science, COMSATS University Islamabad, Lahore Campus, Lahore 54000, Pakistan; aksam.iftikhar@gmail.com; 5 School of Artificial Intelligence, Guilin University of Electronic Technology, Guilin 541004, China

**Keywords:** glioma, computational pathology, cancer grades, texture, machine learning

## Abstract

Cancer pathology reflects disease progression (or regression) and associated molecular characteristics, and provides rich phenotypic information that is predictive of cancer grade and has potential implications in treatment planning and prognosis. According to the remarkable performance of computational approaches in the digital pathology domain, we hypothesized that machine learning can help to distinguish low-grade gliomas (LGG) from high-grade gliomas (HGG) by exploiting the rich phenotypic information that reflects the microvascular proliferation level, mitotic activity, presence of necrosis, and nuclear atypia present in digital pathology images. A set of 735 whole-slide digital pathology images of glioma patients (median age: 49.65 years, male: 427, female: 308, median survival: 761.26 days) were obtained from TCGA. Sub-images that contained a viable tumor area, showing sufficient histologic characteristics, and that did not have any staining artifact were extracted. Several clinical measures and imaging features, including conventional (intensity, morphology) and advanced textures features (gray-level co-occurrence matrix and gray-level run-length matrix), extracted from the sub-images were further used for training the support vector machine model with linear configuration. We sought to evaluate the combined effect of conventional imaging, clinical, and texture features by assessing the predictive value of each feature type and their combinations through a predictive classifier. The texture features were successfully validated on the glioma patients in 10-fold cross-validation (accuracy = 75.12%, AUC = 0.652). The addition of texture features to clinical and conventional imaging features improved grade prediction compared to the models trained on clinical and conventional imaging features alone (*p* = 0.045 and *p* = 0.032 for conventional imaging features and texture features, respectively). The integration of imaging, texture, and clinical features yielded a significant improvement in accuracy, supporting the synergistic value of these features in the predictive model. The findings suggest that the texture features, when combined with conventional imaging and clinical markers, may provide an objective, accurate, and integrated prediction of glioma grades. The proposed digital pathology imaging-based marker may help to (i) stratify patients into clinical trials, (ii) select patients for targeted therapies, and (iii) personalize treatment planning on an individual person basis.

## 1. Introduction

**Figure 1:**
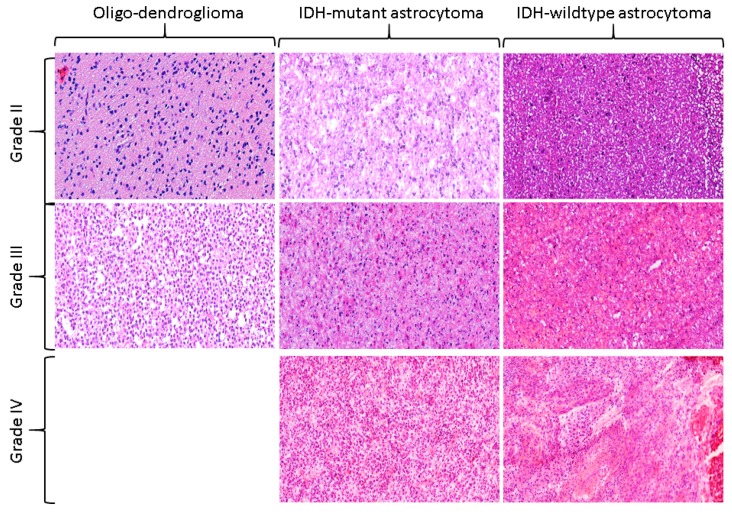
The World Health Organization (WHO) classification of cancer grades. IDH-wildtype astrocytoma is characterized by the absence of both IDH and 1p/19q mutation status, IDH-mutant astrocytoma is defined by the presence of IDH but the absence of 1p/19q, and oligodendroglioma is defined by the presence of both IDH and 1p/19q. Cancer grades are then assigned based on the histologic descriptors of mitotic activity, microvascular proliferation, and pseudopalisading necrosis. The images were acquired using 20× magnification factor.

Gliomas are major malignant tumors of brain originating from glial cells and show huge heterogeneity at the molecular, histological, and imaging levels across and within the same tumors. Gliomas also exhibit a variable proliferation level, which leads to several challenges at the diagnostic and therapeutic fronts [1,2]. Conventionally, diffuse gliomas are classified into astrocytic, oligodendroglial, and mixed oligodendroglial-astrocytic types, and are considered as grade-II (low-grade), grade-III (anaplastic), and grade-IV (glioblastoma) according to World Health Organization (WHO) classification of gliomas [3,4]. For the grading of diffuse gliomas, the histologic features of mitotic activity, microvascular proliferation, and necrosis are used [5]. For a diffuse astrocytoma that does not have any of these features, a diagnosis of low-grade glioma (WHO grade-II) is rendered. The astrocytoma showing elevated mitotic activity is diagnosed as anaplastic astrocytoma (WHO grade-III), even though a specific cutoff of mitotic activity has not been endorsed, thereby leading to some inconsistencies in the grading across different institutions. The presence of necrosis and/or microvascular proliferation leads to a diagnosis of glioblastoma (WHO grade IV). Most of the time, necrosis in glioblastoma comprises of irregular, serpiginous foci surrounded by densely packed, slightly radially oriented tumor cells, and the phenomenon is defined by the term pseudopalisading necrosis. Figure 1 illustrates this taxonomy, where grade II-IV are shown under the umbrella of various molecular subtypes such as Oligodendroglioma, IDH-mutant astrocytomas, and IDH-wildtype astrocytomas. Grade-II-III are referred to as low-grade gliomas (LGG) and grade-IV is referred to as high-grade glioma (HGG) throughout the text.

Until recently, the histologic diagnosis performed on histochemically, especially hematoxylin and eosin, stained sections was the gold standard for glioma grading, conveying prognosis and paving the way for further patient management. Measures provided by a microscopic assessment of the stained sections are, therefore, critical for these cases; the microscopic assessment reveals valuable information critical to disease diagnosis and prognosis [6,7]. Such phenotypic information may encompass collective information represented by the underlying genomic biomarkers and aggressiveness of the disease. Therefore, analysis of these specimens is a widely adopted practice in the medical community. However, microscopic evaluation of surgical or biopsy specimens by trained histopathologists is, in general, of somewhat limited help for rendering a precise diagnosis, mainly in those cases when sample size is limited, is inadequate for testing, or the tissue is fragmented. Moreover, the manual analysis is a highly subjective, tedious, and non-repeatable process owing to the tiresome nature of the analysis and requirement of high-demanding expertise.

Computational pathology is a domain of studying the microscopic images of a tissue specimen to reveal profound disease characteristics [8]. Computational techniques overcome the limitations posed by manual assessment and also provide a secondary opinion to histologists [6,7]. Digitization of tissue slides and the computational techniques developed on those facilitate the: (i) transfer of digital pathology images between different locations for the purposes of education, research, and diagnosis, and most importantly (ii) the solicitation of remote pathology consultation without the need for physically shipping the glass slides around. The digitization process also has the potential to reduce the need for storing glass slides in hospitals and eradicates the risk of glass slides getting broken, misplaced, or lost, though the digital data also processes its risk of being lost in some cases.

One of the pioneering studies performed a deep learning analysis on digital pathology images of gliomas, and confirmed better prognostication when pathology images and genomic biomarkers (*IDH*, *1p/19q*) were used together [9]. Later studies [10] obtained imaging features from pathology images in patients with gliomas and employed deep-learning models. Several histology features, such as pseudopalisading necrosis, geographic necrosis, and inflammation, were found to be associated with the overall survival of gliomas, leading to an accuracy of 90% [11]. Histology features, when combined with radiology features, even further improved prognostication in gliomas [12].

The objective of this study is to develop computational approaches to test the hypothesis that gliomas can be stratified into LGGs and HGGs using *ex vivo* digital pathology images. Such approaches, if they reliably detect the cancer grade using computational methods at the initial presentation of the disease, will help stratification of patients for current and upcoming therapeutic clinical trials. We have employed quantitative feature extraction methods and advanced machine learning for the analysis of computational pathology images. We hypothesize that the quantification of subtle, yet important and spatially complex, histology features as extracted from *ex vivo* digital pathology images is informative and leads to stratifying LGGs and HGGs with sufficient sensitivity and specificity. The main contributions of our study are: (i) to exploit the rich phenotypic information present in histology images through multivariate pattern analysis methods for prediction of glioma grades, and this is the first ever study to use digital pathology images for cancer grading; (ii) to perform robust statistical analysis on all the individual features and their combinations via multiple permuted iterations; and (iii) to validate the model on a large multi-institutional cohort of 735 images.

## 2. Results

**Figure 2:**
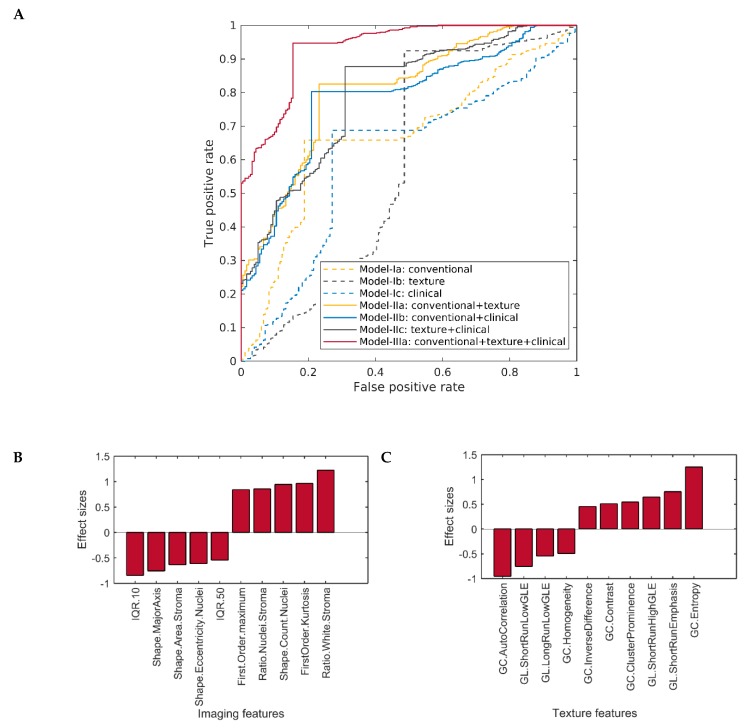
(**A**) Receiver operating characteristics (ROC) curves of one-layer and two-layer models in comparison with the three-layer model based on the predictions of the proposed model. (**B**) Discriminatory capability of conventional imaging features selected by model-1a, and (**C**) discriminatory capability of texture features selected by model-Ib. The discriminatory capability of the features is shown in terms of effect size; the higher the value of effect size, the more discriminative the feature is. The positive effect size indicates the value of the feature to be higher in high-grade gliomas (HGGs), and negative effect size shows the value of the feature to be lower in lower-grade gliomas (HGGs). GL = gray-level run-length matrix, GC = gray-level co-occurrence matrix, GLE = gray-level emphasis, IQR = inter-quantile range.

Three different feature types, including conventional imaging, texture, and clinical features, and their combinations were used for classification via linear kernel of support vector machines (SVM). The dataset is described in Table 1, and the performance of our classification schemes in predicting cancer grades is presented in Table 2. The quantitative results were obtained for all the models and are reported in the tables. The receiver operating characteristic (ROC) curves for each of our predictive schemes in the stratification of patients based on their cancer grade are also shown in Figure 2.

### 2.1. Distribution of the Study Cohort

The study cohort includes 735 formalin-fixed, paraffin-embedded glioma specimens of digital pathology images obtained from the Cancer Genomic Atlas via the Genomic Data Commons (https://gdc.cancer.gov/) for low-grade-gliomas (TCGA-LGG) and glioblastoma (TCGA-GBM) projects [13]. The images were histopathologically diagnosed with different cancer grades, ranging from grade II to the most lethal, grade IV (Table 1). The criteria to include patients in the study were availability of: (i) digital pathology images, (ii) avoidable staining-related artifacts, and (iii) histopathological cancer grade. The cancer grade was downloaded from clinical records provided by The Cancer Imaging Archive (TCIA). Patient identifiers and corresponding records are given in Supplementary material (Supplementary Table S1).

### 2.2. Classification Performance of One-Layer Predictive Models in Detecting Histological Cancer Grade

In the conventional imaging features-based model (model-Ia), the forward feature selection algorithm identified 17 important features to be predictive of cancer grade. Figure 2B shows the top-most conventional features ranked by their discriminatory capability (in terms of effect size) for grade prediction. Based on these features, the model-Ia stratified LGGs and HGGs with 73.14% accuracy (sensitivity: 72.84%, specificity: 74.05%). In the texture features based model (model-Ib), the forward feature selection algorithm identified 11 relevant features to predict cancer grade. Figure 2C shows the top-most texture features ranked by their discriminatory capability (in terms of effect size) for grade prediction. The rest of the relevant conventional and texture features are given in Supplementary Material Table S2. Based on these features, the model-Ib stratified LGGs and HGGs with 75.12% accuracy (sensitivity: 75.30%, specificity: 74.53%). Feature selection was not performed among clinical features (model-Ic) owing to their limited dimensionality, thereby leading to an accuracy of 69.54% (sensitivity: 67.19%, specificity: 76.72%) (Table 2).

### 2.3. Classification Performance of Multi-Layer Predictive Models in Detecting Cancer Grade

On the basis of the selected features in the two-layer models, model-IIa, combining conventional and texture features, stratified patients from LGGs to HGGs with an accuracy of 82.51% (sensitivity: 84.27%, specificity: 77.10%). The other two-layer models, such as model-IIb combining conventional and clinical features, and model-IIc combining texture and clinical features, yielded a slightly lower classification performance of 79.86% (sensitivity: 79.86%, specificity: 75.57%) and 80.90% (sensitivity: 82.47%, specificity: 76.08%), respectively. The three-layered model led to an accuracy of 91.48% (sensitivity: 93.47%, specificity: 85.36%) when all the feature types were collectively used to predict cancer grade (Table 2).

### 2.4. Comparison of the Classification Performance of Different Predictive Models in Detecting Cancer Grade

Table 3 shows the difference in the performance of different models. The performance difference was calculated in terms of area-under-the-curves (AUCs) of the model by running the proposed model in 1000 iterations. The difference in the AUCs of two models was calculated across 1000 permutations, and the average, 95% confidence interval values, and *p*-value were calculated. This analysis provides a robust estimate of the performance improvement achieved by using 2-layer and 3-layer features compared to 1-layer features. No significant difference was noted in the performance of conventional (model-Ia), texture (model-Ib), and clinical (model-Ic) features. Among the two-layer models, model-IIa combining conventional and texture features showed non-significant improvement in grade prediction compared to all the other two-layer models, such as model-IIb combining clinical and conventional imaging features, and model-IIc comprising texture and clinical profiles. The two-layer features when compared with one-layer features, the clinical profile when combined with texture features (model-IIc), and conventional imaging features (model-IIb) showed a non-significant trend toward improved grade prediction, when compared with model-Ic trained on clinical features only (*p* = 0.109 for model-IIb, *p* = 0.102 for model-IIc). On the contrary, the model-IIa combining conventional imaging and texture showed significant improvement in grade prediction, as compared with model-Ia with conventional imaging (*p* = 0.045), model-Ib with texture features (*p* = 0.048), and model-Ic with clinical features (*p* = 0.032).

**Figure 3:**
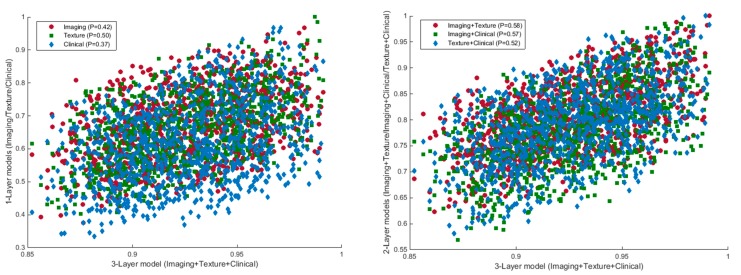
Scatter plots of the AUCs of one-layer (left) and two-layer models (right) in comparison with the three-layer model based on the predictions in 1000 different iterations of the algorithm.

In model IIIa, the texture, clinical, and conventional imaging features yielded significant improvement in grade prediction as compared with one-layer (*p* = 0.002, 0.001, and 0.001 for model-Ia, model-Ib, and model-Ic, respectively) and two-layer models (*p* = 0.004, 0.009, and 0.010 for model-IIa, model-IIb, and model-IIc, respectively). A moderate correlation existed between the performance of a three-layer model and one-layer (*p* = 0.42 for conventional imaging, *p* = 0.50 for texture, *p* = 0.37 for clinical) and two-layer models (*p* = 0.58 for imaging+texture, *p* = 0.57 for imaging+clinical, and *p* = 0.58 for texture+clinical) across different permuted runs (Figure 3).

## 3. Discussion

**Figure 4:**
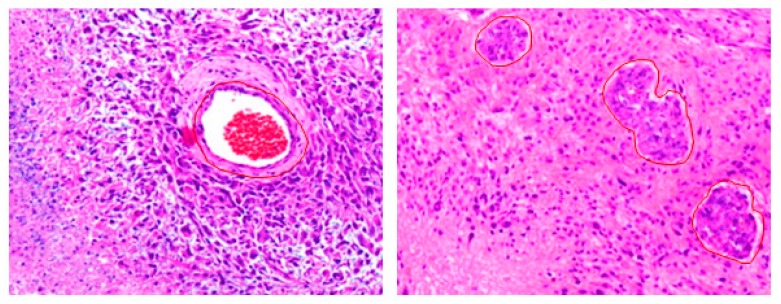
Glioblastoma (grade-IV) tumors. Left: An island of viable tumor cells encircling the blood vessels in a large necrotic focus. Right: Tumor is experiencing endothelial proliferation. The image shows glomeruloid vessels and endothelial multilayering as a result of endothelial hyperplasia. These changes are driven by Vascular Endothelial Growth Factor.secreted by the tumor in response to hypoxia. The images were acquired using 20× magnification factor.

**Figure 5:**
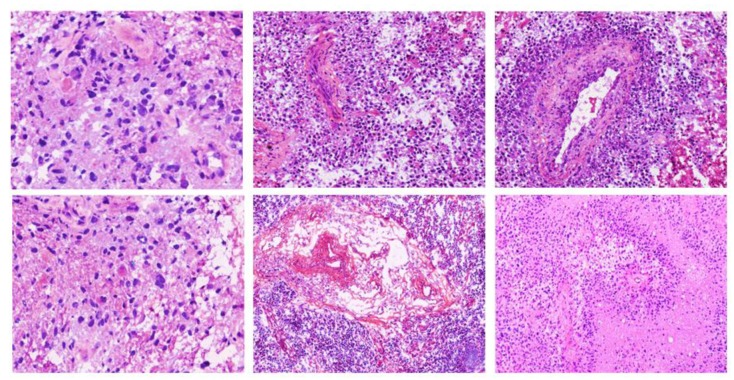
Images showing nuclear pseudopalisading, which is defined as the aggregation of tumor cells around the periphery of the necrotic areas, increased mitotic activity, and vascular proliferation. Pseudopalisading necrosis and vascular proliferation are the two important hallmarks of glioblastoma. The images were acquired using 20× magnification factor.

Alterations in gene expression implore changes at the vascular and structural level in the phenotype of tissue that sequentially can be detected on the particular imaging modality under observation. For example, morphology of the tumor as seen in tissue slides mimics the aggregate effect of molecular alterations/grade/aggressiveness in tumorous cells [9,10]. Figure 4 and Figure 5 show some example images of glioblastomas and its various hallmarks such as pseudopalisading necrosis, vascular proliferation, endothelial proliferation, etc. These histological characteristics help distinguishing different tumor grades. Hence, having a panel of computational tools exploiting the information present in histology images [14,15] is likely to improve our ability to predict the outcome of interest and, hence, to target the right patients with the right treatments, and to monitor response over the course of the disease.

In this article, we employed advanced pattern analysis methods in a cohort of glioma patients that have undergone *ex vivo* digital pathology imaging. We identified a pathology-based imaging signature of the histologic grade of glioma patients, and studied the top-most distinctive pathologic features. Most importantly, the proposed imaging signature was a derivative of a comprehensive and diverse panel of morphological and physiological characteristics of the tumors extracted from pathologic images. In particular, we identified 17 conventional imaging features and 11 advanced texture features extracted from digital pathology images based on forward feature selection and observed that radiomic phenotyping via texture features had incremental predictive value over clinical and conventional imaging features in gliomas.

Existing literature has shown the prognostic and predictive potential of pathologic features in glioma patients [16,17,18], in addition to other cancer types [19]. One of the pioneering studies performed deep learning analysis on digital pathology images of gliomas, and showed improved prognostication when pathology images and genomic biomarkers (*IDH*, *1p/19q*) were used together [9]. A study by Iftikhar et al. [10] employed deep-learning models to exploit several histology features, such as pseudopalisading necrosis, geographic necrosis, and inflammation, which were found to be associated with overall survival. On a related note, the addition of radiomic features extracted from MRI also showed improved prediction of survival compared with those of conventional radiologic (relative cerebral perfusion, apparent diffusion coefficient) measures [17]. Recent studies revealed that the combination of MRI radiomics features with clinical and genetic features improved prognosis on glioma patients when evaluated against the models trained on clinical and genetic features alone [18,20,21,22]. Our investigation builds on the described previous work on digital pathology images by implementing comprehensive quantitative analysis of pathology images in terms of morphological, texture, statistical, signal strength, and clinical features from a large cohort of glioma patients.

The 28 imaging features (17 conventional imaging + 11 texture) identified by our analyses to be most predictive of cancer grade relate to the underlying pathophysiology of gliomas, characterized by the shape of tissue components such as the stroma and nuclei, texture indicating chromatin content inside the nuclei, and the signal strength measures. Morphological and heterogeneity aspects of tumors were summarized in terms of appearance related features (e.g., volume and shape) of nuclei and texture measures in pathology images. In our model, the tumors that were predicted to be LGGs had lower values of contrast and entropy, which is suggestive of reduced spatial heterogeneity, and higher values of correlation and homogeneity, which is suggestive of an elevated spatial homogeneity. Moreover, nuclei in LGGs had higher circularity, smaller volume (size, major- and minor-axis), and regular (sharped) edges, pointing towards the coherent and properly organized structure of the tissue. Our findings are consistent with existing studies on pathology image analysis of other cancers where high-grade tumors have shown lower circularity and larger sizes of nuclei, and elevated spatial heterogeneity and reduced spatial homogeneity [23,24]. These individual features provide discriminatory information, but are not sufficient independently to predict cancer grade. The synergistic use of multiple features, however, yields reasonable cancer grading on an individual patient basis, thereby emphasizing the potential of multivariate methods.

Our study is most likely to be immediately translatable to routine clinical settings and to contribute to precision medicine owing to the use of standard imaging modalities being routinely acquired in almost all institutions. Moreover, the proposed approach considering its generic pipeline may be applicable to other brain tumors after prospective validation. We believe that a rich set of pathology features, and the machine learning signatures derived from these, will enhance our understanding of gliomas and can contribute to precision diagnostics. The proposed method facilitates the real-time transmission of predictive indices calculated on information-rich digital pathology images between different facilities for research, diagnostics, and tutoring purposes. This is mainly suitable for acquiring second-opinion on difficult cases and the option to provide remote consultation without physically shipping tissue slides across different facilities. The method may also advance clinical workflow by minimizing the requirement of storing glass slides in bio-banks of pathology departments and decreasing the risk of glass tissue slides getting damaged. In contrast to costly molecular based assays that not only destroy the tissue, but assess molecular markers from a tiny fraction of the tumor, thereby underestimating tumor heterogeneity, these pathology imaging based companion diagnostic tools could be made available at very reduced price, and could facilitate characterization of the heterogeneity of brain tumors across the entire breadth of the tissue specimen. These tools can help in patient stratification into appropriate treatments, and in the identification of patients with relatively highly heterogeneous tumors, who would benefit from more extensive histopathological and molecular analysis through multiple samples, as well as by combination treatments.

There were several limitations to our study. First, ROI in digital pathology images were semi-automatically outlined, which involved a computer algorithm to find the ROIs and then an expert to make sure that each ROI is free of artifacts. This process might depend on user-bias, and it is also time-consuming. On the other hand, fully automated delineation of ROIs would further automate the workflow, reduce user bias, and facilitate multi-institutional large-scale studies. The use of retrospective dataset is another important limitation of our study; a comparison of the output of our algorithm with cancer grades of a prospective dataset would further validate our model. Moreover, this approach could be applicable clinically if validated on larger volumes of clinical data. This work can be extended along various lines: 1) detailed analysis of the discriminative features that the classification model selects in various cross-validation loops, 2) ensemble methods to exploit different feature types, and 3) to correlate selected histology features with clinical, genetic, and radiology features.

## 4. Methods

**Figure 6:**
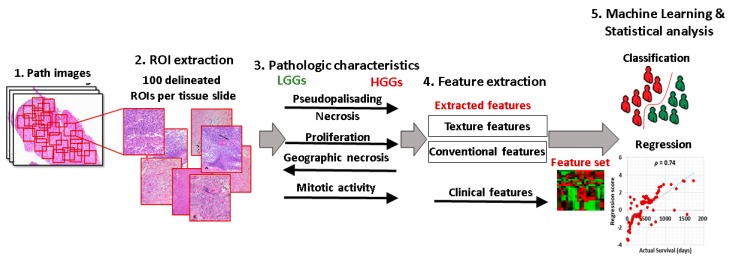
Schematic of the proposed method.

Figure 6 shows overall schematic of the proposed method. In region annotation (Step 2), a set of 100 patches was extracted from each whole slide digital pathology image. In Step 4, images were preprocessed, and a variety of conventional imaging and texture features were extracted. Step 5 was concerned with the incorporation of imaging features into the statistical frameworks and training machine learning models on top of these features.

### 4.1. Data Curation

Digital pathology images were obtained for each patient, and regions of interest (ROI) were delineated. A set of 100 ROIs each comprising 1024 × 1024 pixels that had a viable tumor area with vivid histopathologic features were extracted from images. The patches were selected in a way to make sure that they cover at least 50% of the tissue area (Figure 6, Step 2). All the patches were manually checked for artifacts, and the images free of all types of artifacts were chosen. The images were then converted to gray-scale using Matlab routines before further image processing.

### 4.2. Image Processing and Feature Extraction

All the pathology images were manually checked for artifacts, and the images free of all types of artifacts were chosen. Two types of features, including conventional imaging and texture features, were extracted. The conventional imaging features were comprised of statistical features of mean, std. deviation, median, skewness, kurtosis, max./min. intensity, inter-quantile ranges (IQR) at 10,20,…,90, etc., calculated in the complete image, and morphological features of edge sharpness, perimeter, area, eccentricity, convex area, Euler number, orientation, compactness, and length of major- and minor-axes. In order to calculate morphological features, images were converted to binary image clusters using the K-means clustering algorithm, and connected components were generated in the clusters. The components having an area (number of pixels) greater than a certain threshold were retained. Average values of morphological features were then computed by using all the connected components of a cluster.

In addition, a comprehensive set of texture features, including gray-level run-length matrix (GLRLM) [25] and gray-level co-occurrence matrix (GLCM) [26], was extracted to summarize the content and distribution of chromatin within nuclei, thereby leading to 18 features per image. Texture features have been extensively used in the past for developing diagnostic and prognostic indices [27,28]. To obtain these texture features in 2 dimensions, all images were first quantized to 16 gray levels. GLCM and GLRLM were then populated by taking into account four main directions. A neighborhood of 3 × 3 was considered for GLCM. The features were first computed independently for all the directions (0, 45, 90, 135), and then averaged to find their final value. The features extracted from GLCM include contrast, correlation, energy, homogeneity, entropy, cluster shade, cluster prominence, and autocorrelation, and the features extracted from GLRLM include Long-Run-Emphasis, Short-Run-Emphasis, Run-Length-Non-Uniformity, Grey-Level-Non-Uniformity, High-Grey-Level-Run-Emphasis, Low-Grey-Level-Run-Emphasis, Short-Run-High-Grey-Level-Emphasis, Short-Run-Low-Grey-Level-Emphasis, Long-Run-High-Grey-Level-Emphasis, and Long-Run-Low-Grey-Level-Emphasis. Clinical features of age and gender were used as a separate feature type.

### 4.3. Machine Learning and Statistical Analysis

The experiments of the prediction of histopathological cancer grade were performed in the complete dataset by using one feature type at a time. In the case of conventional imaging features, 10-fold cross-validation was performed using ten different sub-experiments, each time using a 10% testing dataset. A SVM classifier [29] with linear kernel was used to predict the cancer grade. The soft margin cost parameter (C) of linear kernel was optimized via 5-fold cross-validation on the training data using a grid search mechanism; C = 2µ, where µϵ (–5,5). To fit the SVM model, feature selection was performed using the SVM forward feature selection using 5-fold cross-validation on the training data. The forward feature selection mechanism of SVM was used to incrementally keep on selecting the features until the performance either reached a steady state or it started to drop.

Other models with clinical and texture features were also trained on the data by using the 10-fold cross-validation mechanism. In order to assess the incremental value of texture and the conventional imaging features for the prediction of glioma grades, seven models were fitted on the given data for grade prediction; these comprised of models with one-layer features (model-Ia, model-Ib, and model Ic), two-layer features (model-IIa, model-IIb, and model-IIc), and three-layer features (model-IIIa). In particular, the models were trained using the following mechanism: model-Ia was trained on conventional imaging features, model-Ib was on the texture features that consisted of measures extracted from GLCM and GLRLM, model-Ic was on the clinical profile of the patients that consisted of age and gender, model-IIa was on conventional imaging features and texture features, model-IIb was on clinical and conventional imaging features, model-IIc was on clinical profiles and texture features, and model-IIIa was on clinical, conventional imaging, and texture features.

The scores predicted by the models were used to measure the performance of the models in terms of ROC curve analysis. Every model was applied 1000 times on the dataset, each time using a different permutation of the dataset. The AUCs of individual features and the combination thereof were compared. AUC differences were calculated by using the 1000 AUCs calculated in 1000 iterations of the same algorithm. The difference in the performance of the models was considered to be statistically significant when the 95% confidence interval (CI) estimated as the range between the 2.5th and the 97.5th percentile of the difference in AUC did not include zero.

## 5. Conclusions

We proposed a computational method that exploits advanced pattern analysis methods for grade prediction in gliomas using digital pathology images. The results of the proposed method on a dataset comprising 735 subjects are quite encouraging and prove the effectiveness of the proposed method. The proposed method overcomes various limitations, such as high subjectivity and the tedious and non-repeatable nature of manual analysis of histopathology specimens. In conclusion, our results suggest that radiomic phenotyping via texture features improves grade prediction when combined with conventional imaging features and clinical profiles of the patients and, thus, has potential to serve as a practical pathology imaging-based biomarker.

## Figures and Tables

**Table 1:** Clinical and genomic profile of 735 glioma patients involved in the study.

Characteristics	Complete Dataset	LGGs		HGGs
		Grade-II	Grade-III	Grade-IV
No. of patients	735	181 (24.63%)	205 (27.89%)	349 (47.48%)
**Median overall survival**	761.26	1128	842.23	532.45
**No. of deaths**	385 (52.38%)	24 (13.26%)	59 (28.78%)	302 (86.53%)
**Age**	49.65	40.37	45.687	56.79
**Gender**				
Male	427 (58.10%)	99(54.70)	116 (56.59%)	212 (60.74%)
Female	308 (41.90%)	82 (45.30%)	89 (43.41%)	137 (39.26%)
**IDH mutation**				
Wildtype	333 (52.61%)	14 (7.82%)	57 (27.80%)	262 (93.91%)
Mutant	330 (52.13%)	165 (92.18%)	148 (72.20%)	17 (6.09%)
NA	72	2	0	70
**1p19q mutation**				
Wildtype	201 (60.91%)	96 (53.04)	88 (42.93%)	17 (100%)
Mutant	129 (39.09)	69 (38.12)	60 (29.27%)	0

Table entries are either means or the number of patients (percentage distribution in parentheses). IDH: isocitrate dehydrogenase; 1p/19q: deletion of chromosome arm 1p and/or 19q. Percentage for IDH calculated using only the number of patients having the corresponding status available.

**Table 2:** Quantitative results for grade prediction in gliomas. The experiments were performed separately for one-layered models, including model-Ia (imaging features), model-Ib (texture features), and model-Ic (clinical features); for two-layered models, including model-IIa (imaging+texture features), model-IIb (imaging+clinical features), and model-IIc (texture+clinical features); and for three-layered models, including model-IIIa (imaging+texture+clinical features). AUC=area-under-the-curve.

Classification Models	Accuracy	Sensitivity	Specificity	AUC (95% CI)
**One-Layer-Models**				
Model-Ia: Imaging	73.14	72.84	74.05	0.669 (0.477, 0.868)
Model-Ib: Texture	75.12	75.30	74.53	0.653 (0.465, 0.858)
Model-Ic: Clinical	69.54	67.19	76.72	0.610 (0.407, 0.869)
**Two-Layer-Models**				
Model-IIa: Imaging+Texture	82.51	84.27	77.10	0.806 (0.671, 0.937)
Model-IIb: Imaging+Clinical	79.86	81.27	75.57	0.782 (0.642, 0.937)
Model-IIc: Texture+Clinical	80.90	82.47	76.08	0.710 (0.479, 0.936)
**Three-Layer-Models**				
Model-IIIa: Imaging+Texture+Clinical	91.48	93.47	85.36	0.927 (0.872, 0.981)

**Table 3:** Comparison of the performance of the models measured by difference in the area-under-the-curve (AUCs) across 1000 permuted runs. Each entry in the table has *p*-value, average difference of AUC of the models, and confidence interval of 1000 AUCs.

Classification models	Ia: Imaging	Ib: Texture	Ic: Clinical	IIa: Imaging+Texture	IIb: Imaging+Clinical	IIc: Texture+Clinical
**Ia:Imaging**	---	0.530, −0.01 (−0.27, 0.25)	0.636, −0.05 (−0.34,0.25)	---	---	---
**Ib:Texture**	---	---	0.598, −0.05 (−0.31,0.25)	---	---	---
**IIa:Imaging+Texture**	0.045, 0.09 (−0.08,0.34)	0.048, 0.10 (−0.06,0.35)	0.032, 0.20 (−0.06,0.42)	---	0.606, −0.02 (−0.19,0.15)	0.562, −0.01 (−0.17,0.15)
**IIb:Imaging+Clinical**	0.173, 0.12 (−0.11,0.34)	0.123, 0.13 (−0.09,0.34)	0.109, 0.17 (−0.09,0.41)	---	---	0.457, 0.01 (−0.17,0.19)
**IIc:Texture+Clinical**	0.146, 0.13 (−0.09,0.34)	0.117, 0.14 (−0.09,0.33)	0.102, 0.18 (−0.08,0.41)	---	---	---
**IIIa:Imaging+Texture+Clinical**	0.002, 0.26 (0.07,0.43)	0.001, 0.27 (0.09,0.44)	0.001, 0.32 (0.08,0.50)	0.004, 0.12 (0.01,0.23)	0.009, 0.14 (0.02,0.27)	0.010, 0.13 (0.02,0.27)

## Supplementary Materials

The following are available online at https://www.mdpi.com/2072-6694/12/3/578/s1, Table S1: Dataset used in the study, Table S2: Effect sizes of the discriminatory features.
