# Supplementary material for: Glioma Grading via Analysis of Digital Pathology Images Using Machine Learning
Source: Cancers (Basel). 2020 Mar 2;12(3):578. doi: 10.3390/cancers12030578 (PMC7139732; doi:10.3390/cancers12030578)
Supplement: Supplementary file 1 [file cancers-12-00578-s001.zip › cancers-725793-supplementary REVISED .docx]

Glioma grading via analysis of digital pathology images using machine learning

Saima Rathore, Tamim Niazi, Muhammad Aksam Iftikhar, Ahmad Chaddad

**Table S2.** Important features selected by the model-Ia based on conventional imaging features and by model-Ib based on texture features. The importance of the features is shown in terms of effect size. The positive values indicate the value of the feature to be higher in HGGs and negative values show the value of the feature to be lower in HGGs. GLCM = gray-level co-occurrence matrix, GLRLM = gray-level run-length matrix*.*

| **Feature name** | **Importance value**  **(Effect size)** |
| --- | --- |
| **Conventional imaging features** | |
| Ratio.White.Stroma | 1.223 |
| Firstorder.Kurtosis | 0.965 |
| Shape.Count.Nuclei | 0.945 |
| Ratio.Nuclei.Stroma | 0.858 |
| First Order.Maximum | 0.841 |
| First Order.Skewness | 0.834 |
| Shape.Area.Nuclei | 0.756 |
| Shape.Elongation.Nuclei | 0.642 |
| Shape.Eccentricity.Stroma | -0.322 |
| Shape.Compactness.Nuclei | -0.456 |
| Shape.EdgeSharpness.Nuclei | -0.467 |
| IQR.50 | -0.543 |
| Shape.Eccentricity.Nuclei | -0.612 |
| Shape.Area.Stroma | -0.623 |
| Shape.Major Axis | -0.755 |
| IQR.10 | -0.845 |
| **Texture features** | |
| GLCM.Entropy | 1.254 |
| GLRLM.ShortRunEmphasis | 0.754 |
| GLRLM.ShortRunHighGrayLevelEmphasis | 0.645 |
| GLCM.ClusterProminence | 0.546 |
| GLCM.Contrast | 0.512 |
| GLCM.InverseDifferenceNormalized | 0.456 |
| GLCM.ClusterShade | 0.321 |
| GLCM.Homogeneity | -0.494 |
| GLRLM.LongRunLowGreyLevelEmphasis | -0.544 |
| GLRLM.ShortRunLowGreyLevelEmphasis | -0.754 |
| GLCM.AutoCorrelation | -0.954 |
